# Screen time and early childhood development in Ceará, Brazil: a population-based study

**DOI:** 10.1186/s12889-021-12136-2

**Published:** 2021-11-11

**Authors:** Hermano Alexandre Lima Rocha, Luciano Lima Correia, Álvaro Jorge Madeiro Leite, Márcia Maria Tavares Machado, Ana Cristina Lindsay, Sabrina Gabriele Maia Oliveira Rocha, Jocileide Sales Campos, Anamaria Cavalcante e Silva, Christopher Robert Sudfeld

**Affiliations:** 1 Department of Global Health and Population, Harvard T. H. Chan School of Public Health, Boston, MA USA; 2 Department of Maternal and Child Health, Federal University of Ceará, Rua Professor Costa Mendes, 1608 – 5th floor –, Fortaleza, Ceará CEP: 60430-140 Brazil; 3 Department of Community Health, Federal University of Ceará, Fortaleza, Brazil; 4 Department of Exercise and Health Sciences, University of Massachusetts Boston, Boston, MA USA; 5 Service, Education and Community Integration, University Center Unichristus, Fortaleza, Brazil

**Keywords:** Screen time, Child development, Brazil

## Abstract

**Background:**

Globally, children’s exposure to digital screens continues to increase and is associated with adverse effects on child health. We aimed to evaluate the association of screen exposure with child communication, gross-motor, fine-motor, problem-solving, and personal-social development scores.

**Methods:**

We conducted a population-based, cross-sectional study with cluster sampling among children 0–60 months of age living in the state of Ceará, Brazil. Child screen time was assessed by maternal report and the World Health Organization (WHO) recommendations were used to define excessive screen time exposure. Child development was assessed with the Brazilian Ages and Stages Questionnaire. Generalized linear regression was used to determine the association of screen exposure with developmental outcomes. We also examined the potential non-linear relationship of screen time with development scores using spline analyses.

**Results:**

A total of 3155 children 0–60 months of age had screen time exposure evaluated and 69% percent were identified as exposed to excessive screen time. This percentage of excess screen time increased with child age from 41.7% for children 0–12 months to 85.2% for children 49–60 months. Each additional hour of screen time was associated with lower child communication (standardized mean difference (SMD): -0.03; 95% CI: − 0.04, − 0.02), problem solving (SMD: -0.03; 95% CI: − 0.05, − 0.02) and personal-social (SMD: -0.04; 95% CI: − 0.06, − 0.03) domain scores.

**Conclusions:**

Excess screen time exposure was highly prevalent and independently associated with poorer development outcomes among children under 5 years of age in Ceará, Brazil.

**Supplementary Information:**

The online version contains supplementary material available at 10.1186/s12889-021-12136-2.

## Background

Globally, children’s exposure to digital screens continues to increase [1]. International pediatric societies, including the American Academy of Pediatrics and the Brazilian Society of Pediatrics, have stated that parents should limit child time of screen exposure. According to the World Health Organization (WHO), screen time is not recommended for children under two years of age, and sedentary screen time should be no more than one hour per day for children aged two to four years [2].

Multiple studies have found that increased screen time for children is associated with an increased risk of obesity, attention problems and hyperactivity, sleep problems, unsatisfactory academic performance, and unhappiness [3, 4]. In addition, studies have found that excessive screen time is associated with poor early cognitive and motor development outcomes in children [5, 6]. Greater screen time for children may reduce engagement in interactive activities with other children or adults and may lead to fewer learning opportunities t [6, 7]. Therefore, global increases in screen time for children are of major concern given child development may have long-term effects across the life course including on adult productivity [8]. and income generation [9].

Nevertheless, most of the evidence on the relationship of screen time with child development association comes from high-income countries in North America and Europe. To the best of our knowledge, there are no prior studies that have evaluated the association of screen time with development among children in the context of Latin America. To fill this knowledge gap, we conducted a cross-sectional, population-based study in Ceará, Brazil among children aged 0–60 months, to assess the association of screen time exposure with communication, gross-motor, fine-motor, problem-solving, and personal-social development.

## Methods

### Study design and sample

We analyzed data from the *Pesquisa de Saúde Materno Infantil no Ceará* (PESMIC, *Maternal and Child Health Research in Ceará*) study. Full details of the methods for the PESMIC can be found elsewhere [10]. The PESMIC is a population-based cross-sectional study focused on maternal and child health of preschool children up to 72 months of age living in the state of Ceará, in northeastern Brazil. Ceará is one of the poorest states in Brazil, with a population of 9 million inhabitants living in a semiarid climate. Fortaleza (2.3 million inhabitants) is the capital city and urban commercial center of Ceará. The PESMIC study area also included the rural regions of Ceará, where subsistence farming is predominant.

For this analysis, we used data from the 2017 PESMIC survey which was conducted from August to November 2017. The PESMICs used cluster sampling, based on the Brazilian Institute of Geography and Statistics (IBGE) census tracts and stratification between urban Fortaleza and the rural areas. Census tracts were constructed based on the division of each municipality into geographic regions of variable extension with a stable population of 300 families, and 160 randomly selected census tracts that included a total of 3200 households were sampled. To ensure that the study sample was representative, cities, census tracts, and households were randomly selected. Once a census tract was defined and its corresponding map obtained, the location of a 20 house cluster to be surveyed was determined. The starting point of the cluster (the first home to be visited) was randomly selected utilizing ArcGIS® software, GIS Inc. Households were then visited consecutively in a counterclockwise fashion. Shops and abandoned buildings were excluded and replaced and in the case of absent families, up to three return visits were conducted to complete the survey. All mothers aged 10–49 years old and children aged 0–72 months old were included in the PESMIC study and this analysis is limited to children 0–60 months [10].

### Screen time assessment

In order to assess child screen time exposure, the caregiver was asked to enumerate the total amount of time the children spent watching television, using cellphones or tablets, or playing videogames during a habitual day. The study questions can be found in supplementary Chart 1. Excessive screen time was defined based on the WHO guidelines [2]; excessive screen time for children aged 0–23 months was defined as any screen time while excessive screentime for children aged 24–60 months was defined as more than one hour per day. In addition, we also assessed time of exposure to TV and interactive media (time spent on a touch screen or playing devices, e.g., smartphones, tablets, video games).

### Assessment of child development

Child development was assessed using the Ages and Stages Questionnaire, version 3 (ASQ-3), a screening instrument to detect developmental delays in young children, originally developed by Squires, Bricker and Twombly [11]. The PESMIC study used the Brazilian version of the ASQ-3 that was adapted by Santana, Filgueiras and Landeira-Fernandez (ASQ-BR) [12]. The ASQ-BR is composed of 21 age-based questionnaires, with 30 questions in each questionnaire The ASQ-BR assessed the following five domains::

1. globalmotor coordination, which includes movement and use of arms, body, and legs (rolling, crawling, crawling, sitting, walking, running);
2. fine motor coordination, which includesmovements that require control of the use of hands and fingers;
3. communication, which includes questions related to the child’s speech, listening, and comprehension;
4. problem solving, which includes questions related to how children play with toys and their ability to solve problems;
5. personal-social, which includes skills presented in the child’s interactions with other people and the ability to play alone and with others.

In the ASQ-BR questionnaire, there are three possible responses for each time and the response corresponds to a standard point score. “Yes” is scored 10 points, “Sometimes” is scored 5 points and “Not yet” is scored 0 points [11]. The study interviewers were trained to apply the ASQ-BR for 20 h by medical professionals. In terms of scoring, a child’s domain score was excluded if more than two items were skipped. If one or two items in a domain were skipped, we provided an adjusted score by calculating the average score for the completed items in that area and assigned the average score to the skipped item [11]. We also corrected child for prematurity for children aged less than 24 months by subtracting the number of weeks premature from the child’s chronological age.

### Sociodemographic variables

We also assessed child age, sex, maternal education (years of schooling), and family monthly income through questionnaires that were administered to the mother or head of the household. We also assessed household purchasing power using the *Associação Brasileira de Empresas de Pesquisa* (ABEP) questionnaire. The ABEP assesses the ownership of assets by the family, including cars, washing machines, refrigerators, and other possessions. Based on the ABEP score, household are standardly classified into household purchasing power classes [13].

### Statistical analysis

We analyzed the age- and sex-standardized ASQ-BR scores [14] for children aged five to 60 months of age. For children less than five months of age, we used the US ASQ standards [15]. Children with scores < 2 standard deviations (SDs) below the domain mean were considered to screen positive for developmental delay. We then used sample-adjusted generalized linear models to assess the association of screen exposure time with ASQ-BR domain scores. We analyzed total screen time as the primary exposure of interest, and we also assessed TV screen time and interactive media time as secondary exposures of interest. Multivariable models were constructed based on the WHO nurturing care framework and included the covariates for child gender, household purchasing power class, maternal employment, maternal schooling, and interviewer [16]. We assessed the potential non-linear relationship of screen time with development with restricted cubic splines. We used pairwise deletion for missing data. We also assessed the potential for effect modification of the relationship of screen time with development by child age using interaction terms. All study data were double entered twice using EpiInfo 2000 and analyzed using SPSS Version 23 (SPSS Statistics for Windows, Version 23.0. IBM Inc).

## Results

A total of 3155 children 0–60 months of age were included in the analysis. A summary of the sample characteristics is shown in Table 1. The mean maternal age was 28.2 ± 7.1 years, 72.6% were married or had a common-law marriage partner, and 78.7% were unemployed. The mean household income was 1087.7 ± 1004.7 *reais* (~US$ 250.00), and 54.2% participated in *Bolsa Família*, a national conditional cash transfer program. The children’s mean age was 27.1 ± 17.4 months. The reported mean total time of child screen exposure was 2.6 ± 0.6 h per day, of which 1.5 ± 1.7 h were dedicated to watching television and 0.6 ± 1.2 h to the use of tablets and smartphones. Overall, 69% of the children were identified as having excess exposure to screens based on their age. The proportion of children with excessive screen time increased with child age from 41.7% among children aged 0–12 months to 85.2% among children aged 49–60 months (*p*-value < 0.001).

**Table 1:** Sample characteristics including sociodemographic data, screen exposure and assessment of child development by the ASQ-BR; Ceará, Brazil (*n* = 3155)

Sample characteristics	Mean ± SD	N (%)
***Maternal and household characteristics***		
Age (years) (min-max)	28.2 ± 7.1 (10–49)	
Education (years of schooling)	4.5 ± 2.8	
Having a husband or partner		2233 (72.6)
Maternal unemployment		2219 (78.7)
Monthly household income (Brazilian Reais)^a^	1087.7 ± 1004.7	
Participation in conditional cash transfer program^b^		1709 (54.2)
***Child Characteristics***		
Male gender		1582 (50.1)
Age in months	27.1 ± 17.4	
***Screen time (hours per day)***		
Television	1.5 ± 1.7	
Touch devices	0.6 ± 1.2	
Video game	0.3 ± 0.9	
Total screen time exposure	2.6 ± 0.6	
***Excessive screen exposure by age group***		
0–12 months		327 (41.7)
13–24 months		525 (73.6)
25–36 months		441 (68.3)
37–48 months		381 (76.8)
49–60 months		431 (85.2)
Total sample		2454 (69.0)
***ASQ-BR age-standardized scores by domain***		
Communication	52.2 ± 11.5	
Gross motor	55.4 ± 9.3	
Fine motor	49.7 ± 13.7	
Problem solving	50.7 ± 12.5	
Personal-Social	50.1 ± 11.7	

Note: ASQ-3 = Ages and Stages Questionnaire version 3

^a^US$ 1.00 = 3.17 Brazilian Reais at the time of assessment; ^b^ Receiving conditional cash transfer is a marker for low socioeconomic status

The association of screen exposure time with child development outcomes is presented in Table 2. Each additional hour of total screen time was associated with lower child communication (standardized mean difference (SMD): -0.03; 95% CI: − 0.04, − 0.02), problem solving (SMD: -0.03; 95% CI: − 0.04, − 0.01) and personal-social domain scores (SMD: -0.04; 95% CI: − 0.06, − 0.03). There was no association between total screen time and fine and gross motor scores. We also found that increased television time and interactive media time were similarly associated with decreased communication, problem-solving and personal-social scores (Table 2).

**Table 2:** Results of sample-adjusted generalized linear models* to determine the association between screen time and child development domains based on ASQ-BR

	Child development domains (ASQ-BR)									
Screen time (per additional hour)	Communication		Gross motor		Fine motor		Problem solving		Personal-social	
	SMD (95% CI)	p	SMD (95% CI)	p	SMD (95% CI)	p	SMD (95% CI)	p	SMD (95% CI)	p
Total	-0.03	**< 0.001**	−0.004	0.48	−0.010	0.20	−0.03	**< 0.001**	−0.04	**< 0.001**
	(−0.04, − 0.02)		(− 0.017, 0.008)		(− 0.005, 0.000)		(− 0.004, − 0.01)		(− 0.06, − 0.03)	
TV	− 0.06	**< 0.001**	−0.02	0.15	0.009	0.54	−0.06	**0.01**	−0.09	**< 0.001**
	(−0.08, − 0.04)		(− 0.04, 0.01)		(− 0.021, 0.040)		(− 0.08, − 0.03)		(−0.12, − 0.07)	
Interative media	−0.04	**0.001**	0.005	0.71	0.03	0.14	−0.03	**0.04**	−0.05	**0.007**
	(−0.07, − 0.02)		(− 0.02, 0.04)		(− 0.10, 0.07)		(− 0.07, 0.00)		(− 0.09, − 0.01)	

Note: SMD = standardized mean difference; CI = confidence interval

*Multivariable models with SMD for screen time adjusted for child gender, maternal years of schooling, maternal unemployment, purchase power class, family monthly income and interviewer

**Fig. 1:**
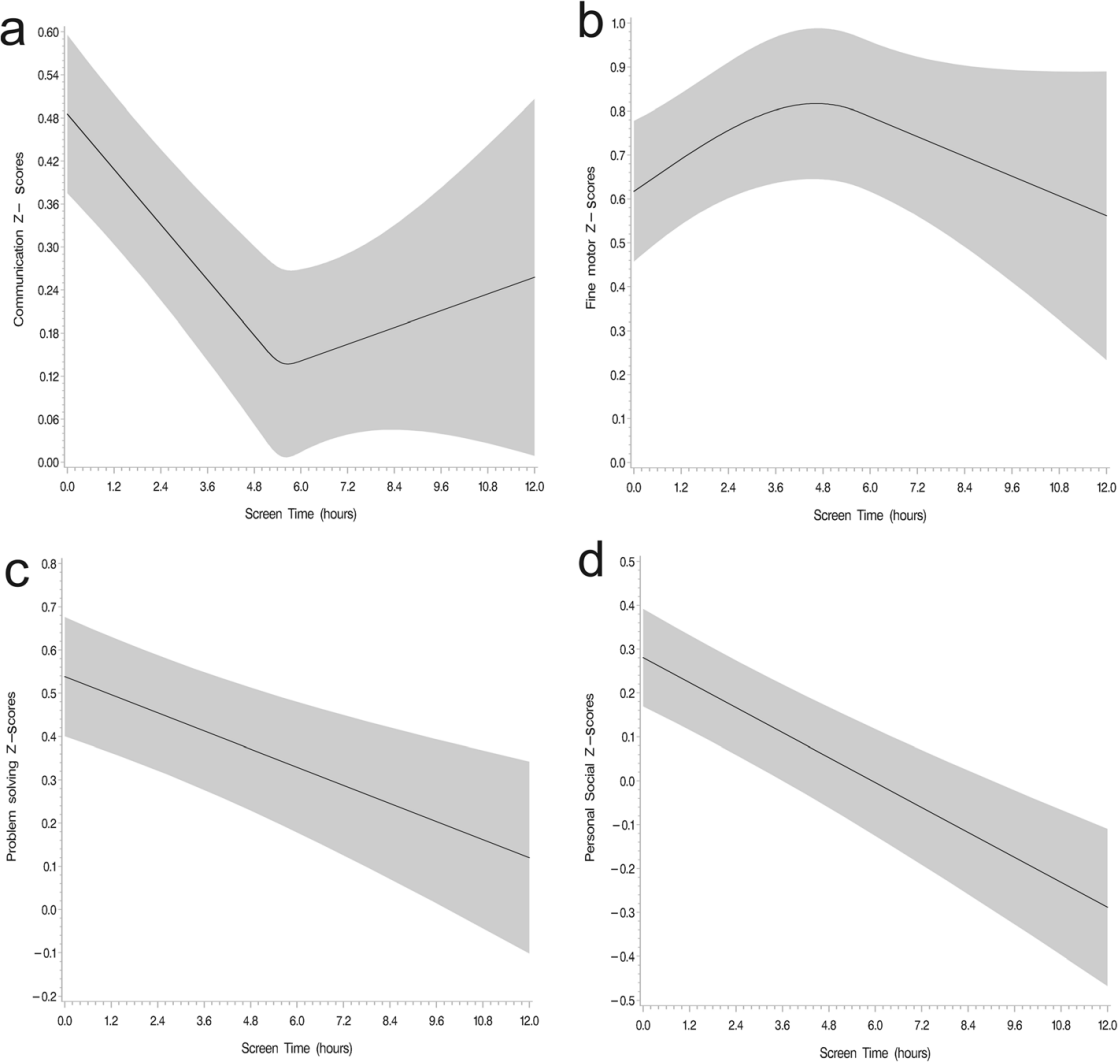
Nonlinear associations between total time of screen exposure with communication (**a**), and fine motor (**b**) scores (p-values for non-linearity < 0.01) and linear associations between time of screen exposure and problem solving (**c**) and personal-social (**d**) ASQ-3 z-scores (*p*-values for linear relation < 0.01) after multivariate adjustment for infant gender, maternal level of schooling, income tertile, ASQ-3 evaluator and maternal employment. Graphs show z-score prediction for girls, mothers that finished elementary school, first income tertile, ASQ-3 evaluator n. 1 and employed mothers. ASQ-3: ages and stages questionnaire version 3

We also examined the potential non-linear relationship of screen time with development outcomes. We found significantly non-linear relationships of total screen time with communication and fine-motor domains (*p*-values for non-linearity < 0.01). Each additional hour of total screen time was associated with lower communication scores up to 6 h per day, after which the relationship appeared to plateau. In contrast, total screen time was associated with increases in fine motor scores to about 4 h, but above 4 h there was a negative association. We found linear relationships for total screen time with problem-solving and personal social domains where each additional hour was associated with lower scores across the observed range of time of screen exposure (*p*-values for linear relationship < 0.01) (Fig. 1). We did not find evidence of effect modification of the relationship of screen time exposure with child development by child age.

## Discussion

In this population-based cross-sectional study in Ceará, Brazil, we found that 69% of children 0–60 months of age had a total screen time in excess of WHO recommendations and the proportion of children with elevated screen time increased with child age. We also determined that increased total screen time was associated with lower child communication, problem solving, and personal-social domains scores.

The prevalence of excessive screen time in our study that used the WHO recommendations is comparable with other studies. For example, in North America, it is estimated that about 50% of children two years of age spend more than one hour/day watching television [17]. In addition, in 2011, 41% of American children up to eight years old had a smartphone at home, while in 2017, this prevalence increased to 95% [18, 19]. Similarly, in Asia, the prevalence of TV exposure greater than one hour/day was 76.7% among two year old children, and in Thailand 90% of two year of children had greater than one hour per day of screen time exposure [20, 21]. Population data on the prevalence of screen time in young children in Latin America in rare. In Brazil, a small study with 180 daycare children in Diamantina, Minas Gerais, found that 48.2% of children aged 2 years old had greater than two hours of screen exposure [22], which is slightly lower than our population-based data.

We also found that increased screen time was negatively associated with child communication, problem-solving, and personal social domain scores after adjustment for potential confounders. The relationship of increased screen exposure with poorer communication and language development, has been well documented in the literature [23]. Excessive exposure to screen time can reduce the time during which the child engages in conversations with adults and older children, which may lead to impaired language development [24]. In addition, the accumulation of visual stimuli and brain exposure to screen images can be harmful and can lead to damage to the frontopolar region of the brain, responsible for language development [25].

We also found that increased screen time was associated with poorer personal-social and problem-solving development in our study. There is some evidence that television watching can influence children’s behavior, which may lead to suboptimal development of personal-social skills. A study carried out in Bangkok, Thailand, found that children that were exposed to adult television programs starting from six months of age were at greater risk for pervasive developmental problems, oppositional defiant behaviors, emotionally reactive problems, aggression, and externalizing behaviors [26]. In addition, a study that evaluated more than 3000 3-year of children in the US reported that children who had greater exposure to television were more likely to exhibit violent behavior [27]. Nevertheless, it is important to note that greater screen time may be a consequence of limited time and ability of mothers and caregivers to engage with their children and therefore the associations may not be biologically related to screen time but rather the result of more limited engagement of parents in play and learning activities [28].

Nevertheless, it is important to note that not all screen time may negatively affect child development. A recent study found screen-mediated activities, such as storytelling, had positive effects on child development during the COVID-19 pandemic [29]. A study carried out in Brazil identified that exposure to high-quality screen time with mediation and parental participation was associated with better child development outcomes [30]. Further, there is evidence that type of screen exposure may differentially be associated with children’s development. For example, a cohort conducted in Australia among 10–11 year old children found that that passive screen time (such as watching television) was associated with worse development while the use of interactive screens (such as cell phones and video games) was positively related to some educational outcomes [31]. However, it is important to note, the relationship of type of screen time with development may not apply to younger children. In our study, we did not have data on the programming or type of media that children were exposed to. Brazil has a low cable television coverage, and the open-access channels have few educational programs for children which may be related to Brazilian laws that prohibit advertising in child-directed content. Therefore, it is important for future research to provide a more detailed assessment of screen exposure and the types of media children are engaged in.

Our study has a few limitations. The cross-sectional design of the study does not allow for the analysis of child development trajectories over time or direct determination of causal associations. In addition, we adjusted for socioeconomic status, maternal education, and other factors, but there may still be residual and unmeasured confounding. Furthermore, while the study was designed to be representative of the child population in the State of Ceará, it might not be generalizable to all children in Brazil or other settings in Latin America.

## Conclusions

Overall, our population-based survey found that 7 out of 10 children 0–60 months of age in Ceará, Brazil had excessive screen time exposure. Increased total time of screen exposure was associated with poorer child communication, problem solving and personal-social development. As a result, research on interventions that aim to reduce child screen exposure and promote child development is needed.

## Supplementary Information

**Additional file 1.** Specific questions used to evaluate screen time exposure

## Data Availability

The datasets used and/or analyzed during the current study are available from the corresponding author on reasonable request.

## Abbreviations

PESMIC: Pesquisa de Saúde Materno Infantil do Ceará
IBGE: Brazilian Institute of Geography and Statistics
LMIC: low- and middle-income countries
SES: Socioeconomic status
SDG: Sustainable development goals

## References

[1] Ribner AD, McHarg G (2021). Screens across the pond: findings from longitudinal screen time research in the US and UK. Infant Behav Dev.

[2] Organization WH. Guidelines on physical activity, sedentary behaviour and sleep for children under 5 years of age: World Health Organization; 2019. https://apps.who.int/iris/handle/10665/311664. 31091057

[3] Stiglic N, Viner RM (2019). Effects of screentime on the health and well-being of children and adolescents: a systematic review of reviews. BMJ Open.

[4] Jackson DB, Testa A, Fox B (2021). Adverse childhood experiences and digital Media use among U.S. children. Am J Prev Med.

[5] Walsh JJ, Barnes JD, Tremblay MS, Chaput J-P (2020). Associations between duration and type of electronic screen use and cognition in US children. Comput Hum Behav.

[6] Madigan S, Browne D, Racine N, Mori C, Tough S (2019). Association between screen time and children’s performance on a developmental screening test. JAMA Pediatr.

[7] Zimmerman FJ, Christakis DA, Meltzoff AN (2007). Associations between media viewing and language development in children under age 2 years. J Pediatr.

[8] Smith JP (2009). The impact of childhood health on adult labor market outcomes. Rev Econ Stat.

[9] Fink G, Peet E, Danaei G, Andrews K, McCoy DC, Sudfeld CR, Smith Fawzi MC, Ezzati M, Fawzi WW (2016). Schooling and wage income losses due to early-childhood growth faltering in developing countries: national, regional, and global estimates, 2. Am J Clin Nutr.

[10] Correia LL, Rocha HAL, Rocha SGMO, et al. Methodology of Maternal and Child Health Populational Surveys: A Statewide Cross-sectional Time Series Carried Out in Ceará, Brazil, from 1987 to 2017, with Pooled Data Analysis for Child Stunting. Annals of Global Health. 2019;85(1). 10.5334/aogh.2299 PMC6997523 30873783

[11] Squires J, Bricker DD, Twombly E (2009). Ages & stages questionnaires.

[12] Santana, C. M., Filgueiras, A., & Landeira-Fernandez, J. (2015). Ages & stages questionnaire–Brazil–2011: adjustments on an early childhood development screening measure. Global pediatric health, 2, 2333794X15610038. 10.1177/2333794X15610038 PMC4784636 27335984

[13] Associação Brasileira de Empresas de Pesquisa (ABEP). São Paulo: Critério de classificação econômica Brasil. 2020.

[14] Filgueiras A, Pires P, Maissonette S, Landeira-Fernandez J (2013). Psychometric properties of the Brazilian-adapted version of the ages and stages questionnaire in public child daycare centers. Early Hum Dev.

[15] Janson H, Squires J (2004). Parent-completed developmental screening in a Norwegian population sample: a comparison with US normative data. Acta Paediatr.

[16] Wertlieb D (2019). Nurturing care framework for inclusive early childhood development: opportunities and challenges. Dev Med Child Neurol.

[17] Saunders TJ, Vallance JK (2017). Screen time and health indicators among children and youth: current evidence, limitations and future directions. Appl Health Econ Health Policy.

[18] Media CS (2013). Zero to eight: Children’s media use in America 2013.

[19] Rideout V. The common sense census: Media use by kids age zero to eight. San Francisco, CA: Common Sense Media. 2017:263–83.

[20] Intusoma U, Mo-Suwan L, Chongsuvivatwong V (2013). Duration and practices of television viewing in Thai infants and toddlers. *Journal of the medical Association of Thailand =*. Chotmaihet thangphaet.

[21] Byeon H, Hong S (2015). Relationship between television viewing and language delay in toddlers: evidence from a Korea national cross-sectional survey. PLoS ONE.

[22] Nobre JNP, Santos JN, Santos LR, Guedes SC, Pereira L, Costa JM, Morais RLS (2021). Determining factors in children’s screen time in early childhood. Ciência & Saúde Coletiva.

[23] Madigan S, BA MA, Anhorn C, Eirich R, Christakis DA. Associations Between Screen Use and Child Language Skills: A Systematic Review and Meta-analysis. JAMA Pediatr. 2020;174(7):665–75. 10.1001/jamapediatrics.2020.0327 PMC7091394 32202633

[24] Chonchaiya W, Pruksananonda C (2008). Television viewing associates with delayed language development. Acta Paediatr.

[25] Takeuchi H, Taki Y, Hashizume H, Asano K, Asano M, Sassa Y, Yokota S, Kotozaki Y, Nouchi R, Kawashima R (2015). The impact of television viewing on brain structures: cross-sectional and longitudinal analyses. Cereb Cortex.

[26] Chonchaiya W, Sirachairat C, Vijakkhana N, Wilaisakditipakorn T, Pruksananonda C (2015). Elevated background TV exposure over time increases behavioural scores of 18-month-old toddlers. Acta Paediatr.

[27] Manganello JA, Taylor CA (2009). Television exposure as a risk factor for aggressive behavior among 3-year-old children. Arch Pediatric Adolesc Med.

[28] Lindsay AC, de Sá Melo Alves A, GVdB V, et al. A qualitative study conducted in the United States exploring the perspectives of Brazilian immigrant fathers about their preschool-age children’s physical activity and screen time. J Public Health. 2021.

[29] Gaudreau C, King YA, Dore RA, Puttre H, Nichols D, Hirsh-Pasek K, Golinkoff RM (2020). Preschoolers benefit equally from video chat, pseudo-contingent video, and live book reading: implications for storytime during the coronavirus pandemic and beyond. Front Psychol.

[30] Nobre JN, Vinolas Prat B, Santos JN (2020). Qualidade de uso de mídias interativas na primeira infância e desenvolvimento infantil: uma análise multicritério. J Pediatr.

[31] Sanders T, Parker PD, del Pozo-Cruz B, Noetel M, Lonsdale C (2019). Type of screen time moderates effects on outcomes in 4013 children: evidence from the longitudinal study of Australian children. Int J Behav Nutr Phys Act.

