# Supplementary material for: Screen time and early childhood development in Ceará, Brazil: a population-based study
Source: BMC Public Health. 2021 Nov 11;21:2072. doi: 10.1186/s12889-021-12136-2 (PMC8582336; doi:10.1186/s12889-021-12136-2)
Supplement: Supplementary file 1 — Additional file 1. Specific questions used to evaluate screen time exposure [file 12889_2021_12136_MOESM1_ESM.docx]

Supplementary Chart 1. Specific questions used to evaluate screen time exposure.

| How many hours a day does * watch TV? |
| --- |
| How many hours a day does * stay on the internet? |
| How many hours a day * is on electronic touch devices on the screen? |
| How many hours a day does * **stay in** electronic games (computer/mobile/video game)? |
